# Temporal Change of Interleukin-6, C-Reactive Protein, and Skin Temperature after Total Knee Arthroplasty Using Triclosan-Coated Sutures

**DOI:** 10.1155/2018/9136208

**Published:** 2018-01-15

**Authors:** Shih-Jie Lin, Fu-Chun Chang, Tsan-Wen Huang, Kuo-Ti Peng, Hsin Nung Shih, Mel S. Lee

**Affiliations:** ^1^Department of Orthopaedic Surgery, Chang Gung Memorial Hospital, Chiayi, Taiwan; ^2^Chang Gung University, Taoyuan, Taiwan; ^3^Department of Orthopaedic Surgery, Chang Gung Memorial Hospital, Linkou, Taiwan; ^4^Bone and Joint Research Center, Chang Gung Memorial Hospital, Linkou, Taiwan; ^5^Department of Orthopaedic Surgery, Chang Gung Memorial Hospital, Kaohsiung, Taiwan

## Abstract

The risk of surgical site infections (SSIs) after total knee arthroplasty (TKA) can never be eliminated. Antimicrobial sutures containing triclosan have been used to decrease SSIs, but whether triclosan-coated sutures are effective with TKA is unclear. Between 2011 and 2012, 102 patients randomly assigned to a triclosan or a control group were prospectively assessed. The incidence of SSI within 3 months of surgery, length of hospital stay, pain scale, functional scores, wound condition, and serum inflammatory markers during hospitalization and within 3 months postoperatively were compared. At the final follow-up, there were 2 patients with superficial infections (3.9%) in the control group but none in the triclosan group. Lower serum IL-6 was detected in the triclosan group at 4 weeks and 3 months. The local skin temperature of the knees—recorded at 3 months using infrared thermography—was lower in the triclosan group than in the control group. More precise analytical measurements are needed to investigate local and systemic complications, especially in the early subclinical stage. This prospective, randomized, open-label clinical trial is in the public registry: ClinicalTrials.gov (NCT02533492).

## 1. Introduction

The risk of complications after total knee arthroplasty (TKA) can never be eliminated because of the risk of surgical site infections (SSIs) [1]. Although the reported incidence of a periprosthetic joint infection (PJI) after TKA is only about 1–3% [1–4], complications such as prolonged wound drainage are associated with a 17–50% incidence of SSI after arthroplasty [5–8].

PJI has devastating effects on patients and healthcare systems. When a PJI is established, it typically requires removal of the prosthesis, extensive debridement, prolonged antibiotic treatment, and reimplantation. However, the reinfection rate associated with reimplantation for a PJI is significantly higher than for those without infections, and this, in turn, is associated with a longer hospital stay, increased risk of comorbidities, and a major economic burden [9–12]. Measures to reduce the incidence of infections are extensively implemented in clinical practice [13]. Triclosan is an antibacterial and antifungal agent that has been widely used in humans for more than 30 years [14]. Triclosan-coated sutures reduce wound complications related to bacterial infections in animal studies and in abdominal, gastrointestinal, and coronary bypass surgery [15–22]. However, its efficacy in preventing post-TKA infections has not been previously investigated.

In this prospective randomized double-blind study, we investigated whether triclosan-coated sutures prevent SSIs after TKA surgery by lowering the risk of local bacterial infection. The primary outcome measure was the incidence of SSIs, and the secondary outcome measures were inflammatory markers (CRP: C-reactive protein; ESR: erythrocyte sedimentation rate; IL-6: interleukin 6), local wound temperature and conditions, and functional scores at given in-hospital and postoperative time points.

## 2. Materials and Methods

### 2.1. Study Design

This prospective double-blind randomized controlled trial is registered at ClinicalTrials.gov (NCT02533492). The Institutional Review Board of Chang Gung Memorial Hospital approved the study protocol (IRB: 101-0352C). All study participants provided written informed consent.

### 2.2. Inclusion and Exclusion Criteria

The inclusion criteria were men and women aged 55–85, diagnosed with degenerative osteoarthritis of the knee, and not having previously undergone surgery to the index knee. Patients with inflammatory arthritis—rheumatoid arthritis, ankylosing spondylitis, infectious arthritis, systemic lupus erythematosus, and psoriatic arthritis—were excluded. Other exclusion criteria were a history of cancer within 5 years before the initial study screening, osteogenesis imperfecta, Paget's disease, neurovascular disease of the lower extremities, liver cirrhosis, an aspartate aminotransferase or alanine aminotransferase level more than twice the maximum normal value at screening, coagulopathy, serum creatinine lower than 35 ml/min at screening, having undergone hemodialysis for renal failure, peripheral arterial occlusive disease, a preoperative international normalized ratio over 1.5 at screening, an American Society of Anesthesiologists physical classification system score over 3, or an immunocompromised condition.

**Figure 1:**
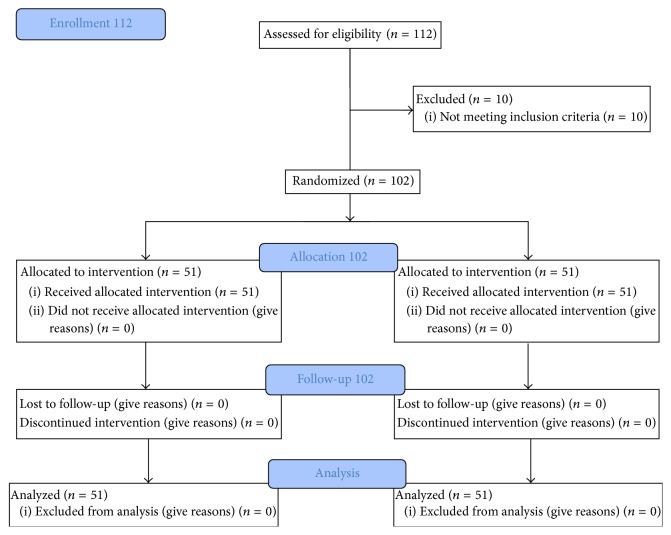
Overview of study design in CONSORT 2010 flow diagram.

Between June 2011 and May 2012, we screened 112 patients scheduled for a unilateral TKA at Chang Gung Memorial Hospital: 102 eligible patients were enrolled and randomly assigned to one of two equal groups of 51 (Figure 1). Group sample sizes of 51 and 51 provided 81% power to detect a difference of 4% between the null hypothesis (that both group means would be 9.0) and the alternative hypothesis (that the mean would be 13.0 with estimated group standard deviations [SDs] of 6.0 and 8.0). Significance (*α*) was set at 0.05 using a two-sided two-sample* t*-test.

One hundred two sets of suture materials (51 sets of triclosan-coated polyglactin sutures [Vicryl Plus, Ethicon, Johnson and Johnson Medical] and 51 sets of plain polyglactin sutures) were placed into 102 separate sealed envelopes consecutively numbered from 1 to 102 based on the randomization protocol and then randomly given to the eligible patients for wound closure. During the study, the allocation of these suture materials was blinded to the patients, the clinical staff, the operating surgeons, and the independent study nurse who prospectively collected all perioperative information and outcome measures. Only the circulating nurse who opened the envelopes and the scrub nurse who handled the suture materials were not blinded, but they were not involved in evaluating the study.

### 2.3. Total Knee Arthroplasty

All TKA surgery used the standard medial parapatellar technique and the standard clinical pathway. Cemented knee prostheses (NexGen LPS-Flex Fixed Bearing Knee; Zimmer, Warsaw, IN) were implanted in all patients, and all patellae were resurfaced. Wound closure was done in three layers after insertion of a closed Hemovac drainage tube. The arthrotomy, fascial layer, and subcutaneous wound were closed using triclosan-coated polyglactin sutures in the triclosan group and plain polyglactin sutures in the control group. The outermost skin edge was stapled to facilitate postoperative wound assessment.

All patients were given systemic antibacterial prophylaxis (cefazolin: 1 g if body weight was <80 kg; 2 g if body weight was >80 kg) 30 to 60 minutes before the skin incision. Postoperatively, each patient was given 3 intravenous doses of cefazolin at 8-hour intervals. The wounds were photographed and assessed by the study nurse and surgeons who were blinded to and independent of the study.

### 2.4. Assessments

An experienced clinician, blinded to group assignment and patients' demographic data, performed all radiographic and clinical assessments.

The demographic characteristics and perioperative laboratory data of the patients were prospectively collected and registered in a database. Samples for preoperative laboratory analysis (hemoglobin, white blood cell count, platelet count, and serum creatinine) were collected on the day before surgery and analyzed using standard clinical methods. Preoperatively, skin condition (surface temperature, digital photo, and image analysis) (ATIR-M301 Infrared Thermal Imaging System, Associated Technology Corporation, Chongqing, Sichuan, PROC), inflammatory markers (CRP, ESR, and IL-6), functional scores (knee range of motion) (KSS: Knee Society Score; SF-12: Short Form 12), and pain (Visual Analogue Scale [VAS]) were assessed and recorded.

On the 1st and 3rd postoperative day during hospitalization, skin condition, functional scores, inflammatory markers, and pain were evaluated. The surgical wounds were photographed and assessed by the study nurse and surgeons who were blinded to and independent of the study.

**Figure 2:**
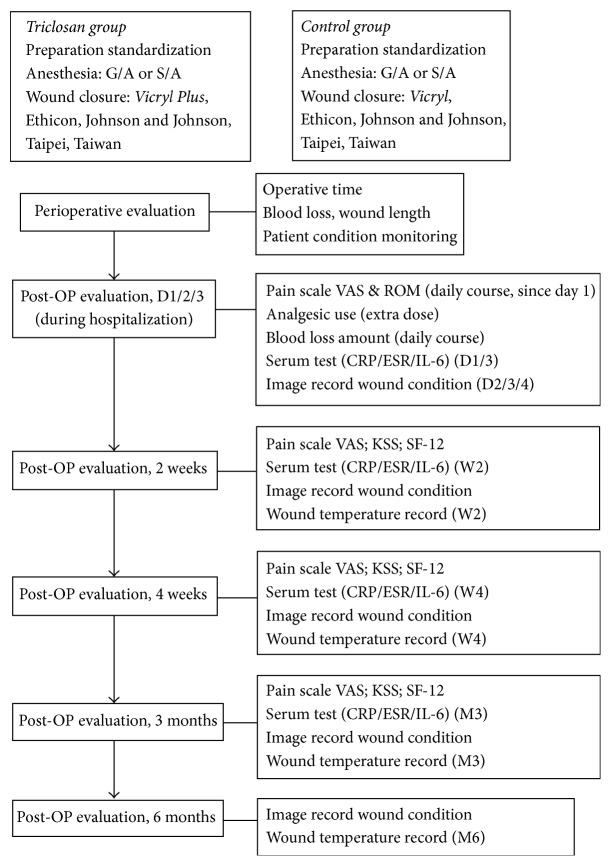
Follow-up protocol.

At 2 weeks, 4 weeks, and 3 months after surgery, skin condition, functional scores, inflammatory markers, and pain were assessed and recorded (Figure 2). An additional wound image and temperature record were performed at postoperative 6 months. This is a relatively noninvasive assessment for inflammation analysis.

The primary outcome measure was the incidence of SSI within 3 months of surgery. The secondary outcome measures included length of hospital stay, pain level, functional scores, wound condition (wound drainage, extent of erythema, local heat, and skin surface temperature), and inflammatory markers during hospitalization and within 3 months postoperatively.

### 2.5. Statistical Analysis

An independent statistician analyzed all data. Continuous variables are presented as means and standard deviations (SDs) and categorical data as percentages and proportions. Differences between groups were analyzed using the independent *t*-test for continuous variables, and *χ*^2^ and Fisher's exact tests for categorical variables. For all tests, significance was set at *p* < 0.05 (two-tailed). SPSS 17.0 (SPSS Inc., Chicago, IL) was used for all data analyses.

## 3. Results

We analyzed the data from 102 patients: 26 men; 76 women; mean age: 70.7 ± 7.4 years (Table 1). There were no significant differences in demographic data between the triclosan and control groups. The triclosan group contained 15 men and 36 women (mean age: 71.3 ± 7.7 years), and the control group contained 11 men and 40 women (mean age: 70.0 ± 7.1 years). The surgical duration was significantly shorter in the triclosan group (125 ± 24 minutes versus 136 ± 34 minutes, *p* = 0.046) (Table 2). There were no significant differences in the length of the incision, blood loss, or 3-month postoperative wound complication rates between the two groups.

**Figure 3:**
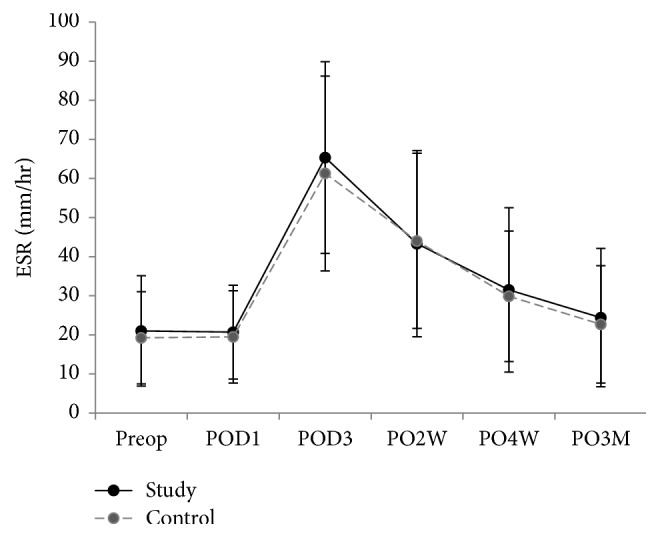
Serum erythrocyte sedimentation rate (ESR) levels. Plot graph showing the mean ESR levels of patients after total knee replacement with triclosan-coated polyglactin sutures (triclosan group) or with plain polyglactin sutures (control group) from preoperative period to 3-month follow-up (error bars represent SDs).

**Figure 4:**
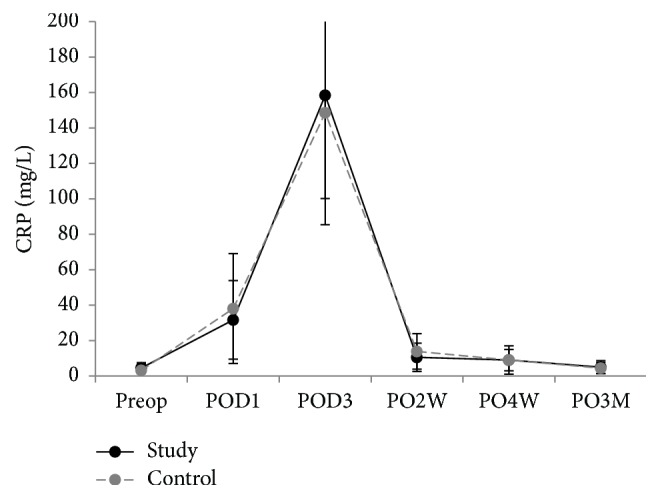
Serum C-reactive protein (CRP) levels. Plot graph showing the mean CRP levels of patients following total knee replacement with triclosan-coated polyglactin sutures (triclosan group) or with plain polyglactin sutures (control group) from preoperative period to 3-month follow-up (error bars represent SDs).

**Figure 5:**
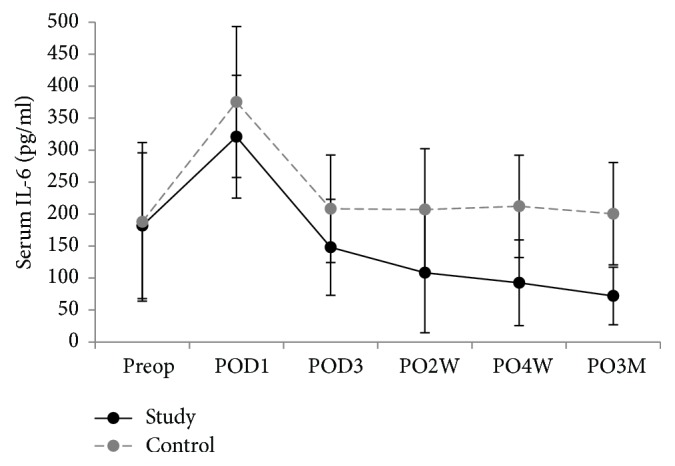
Serum IL-6 levels. Plot graph showing the mean IL-6 levels of patients following total knee replacement with triclosan-coated polyglactin sutures (triclosan group) or with plain polyglactin sutures (control group) from preoperative period to 3-month follow-up (error bars represent SDs).

**Figure 6:**
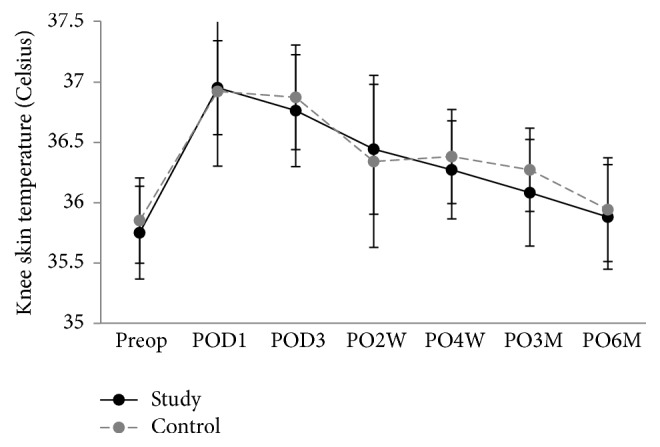
Skin surface temperature. Plot graph showing the mean skin surface temperature levels of patients following total knee replacement with triclosan-coated polyglactin sutures (triclosan group) or with plain polyglactin sutures (control group) from preoperative period to 6-month follow-up (error bars represent SDs).

Inflammatory markers were compared in both groups 6 times: preoperative day 0; postoperative days 1 and 3; weeks 2 and 4; month 3. There was a significantly lower level of IL-6 in the triclosan group than in the control group between week 4 (*p* < 0.001) and month 3 (*p* = 0.05) (Figures 3–5). VAS, KSS, and SF-12 scores were not significantly different except on postoperative day 1, when the VAS score of the triclosan group was higher than that of the control group (8.6 versus 8.1, *p* = 0.017) (Table 3). The knee skin surface temperature in both groups was compared 6 times. There were significantly lower surface temperatures in the triclosan group than in the control group in postoperative month 3 (*p* = 0.022) (Figure 6).

There were 2 cases of superficial surgical site infections (SSIs) in the control group (Table 2). Wound cultures showed that case 1 was positive for* Klebsiella pneumoniae* and case 2 was negative. Both cases were managed with parenteral antibiotics for 1 week (oxacillin and gentamicin for case 1; cefazolin for case 2) and then with oral antibiotics at home for another week. Both cases resolved without further complications. The infection rate was 0% in the triclosan group and 3.9% in the control group (*p* = 0.495). No deep SSIs were found in either group.

## 4. Discussion

We analyzed the efficacy of triclosan-coated sutures for reducing SSIs and preventing deep PJIs after TKA by rigorously evaluating wounds, measuring inflammatory markers, monitoring skin temperature, and assessing functional outcomes. However, because there were only two superficial SSIs in the control group and none in the triclosan group, we were unable to arrive at any conclusions about their protective efficacy against SSIs.

Some studies [23–25] have reported significantly lower wound complication rates with triclosan-coated sutures than with conventional sutures in digestive tract surgery, open vein harvesting of coronary artery bypass surgery, colorectal surgery, and hepatobiliary surgery. Hoshino et al. [26] reported a lower incidence of SSIs in Class II (clean-contaminated) surgical wounds closed with triclosan-coated sutures. Other studies [27–29], however, have reported comparable wound complication rates in head and neck surgery and general pediatric surgical procedures [27–29]. Our review of the literature showed that no similar clinical trials have compared triclosan-coated sutures with conventional sutures in TKA. In our prospective double-blind randomized controlled trial, we restricted our enrolled patients to those with degenerative osteoarthritis of the knee who had not previously undergone surgery to the index knee. Confounding factors might have been reduced because all our patients were treated by the same experienced surgeon, with the same prostheses, with the same surgical technique, and with the same treatment protocol. In addition, patients with conditions that might alter or compromise immune systems were excluded. In the present study, however, we found no significant differences in the efficacy of reducing SSIs in TKA. TKA is a Class I (clean) surgery; thus, the benefits of triclosan-coated sutures to combat microorganisms might not be demonstrated.

CRP, ESR, and IL-6 inflammation levels were analyzed as surrogates for systemic reactions; However, except for IL-6 from 4 postoperative weeks to 3 months, they were not significantly different between the triclosan and control groups. ESR and CRP are reasonably sensitive and acceptably specific post-TKA diagnostic markers for PJI in a selected group of patients [30]. However, plasma levels of these acute-phase proteins might be confounded by intraoperative tissue trauma and physiological inflammation when used to detect early subclinical infections. IL-6 induces and regulates the acute phase of inflammation, which might make it more rapid and sensitive. IL-6 levels peak within 6–12 hours after major surgery and then return to baseline levels within 48–72 postoperative hours [31–35]. We also found that IL-6 levels rapidly declined by the third postoperative day, which is consistent with other reports [36–39]. However, IL-6 in our triclosan group fell to lower than baseline levels at 2 weeks, 4 weeks, and 3 months. But in the control group, IL-6 remained at about baseline level during the follow-up.

This temporal pattern of serum IL-6 was interesting. Studies [40–42] have reported that triclosan causes human health and environmental problems, and the manufacturer announced that, from 2015, triclosan would be phased out of their baby and beauty products. Although we found no significant advantage of the triclosan-coated sutures on lowering the incidence of SSIs after TKA, we hypothesize that the triclosan in the sutures at least prevented an increase in IL-6 levels. This requires confirmation in future studies, however.

There were no significant differences in the mean global or regional surface temperature of the operated knee between the triclosan and control groups during the 6-month follow-up. As with the analysis of early postoperative subclinical inflammation in primary TKA [36, 39, 43], surface temperature changes mirrored the levels of serologic inflammatory markers. It may also have been confounded and masked by the abovementioned factors. Therefore, a more sensitive analytical tool might be necessary to investigate differences in surface temperature and other early-stage subclinical wound complications.

The functional outcomes of the patients in the present study improved in both groups; however, there were no important significant differences between the groups throughout the 3-month postoperative follow-up. Thus, using triclosan-coated sutures to close the wounds of primary TKAs had no significant effect on postoperative clinical outcomes or knee inflammation. Soft-tissue damage during the surgery induced far greater synovial inflammation than what the relatively small amount of triclosan in the antibacterial sutures could counteract.

This study has some strengths. All patients were followed up completely, and comprehensive serological tests, wounds, infrared thermography, and functional outcomes were assessed. The prospective double-blind randomized design of this study also allowed us to independently assess the results of using triclosan-coated sutures on post-TKA surgical wounds.

This study also has some limitations. First, the sample was not large enough to demonstrate the superiority of triclosan-coated sutures in preventing SSIs in TKA. This specific sample size was chosen to attain an 80% power of analysis based on the reported incidence of SSIs after TKA [1–4]. A much larger sample seems to be needed if the occurrence of PJI is taken as the endpoint of analysis; however, it might be unethical not to use antimicrobial sutures to test the hypothesis. Instead, we conducted a noninferiority study, after IRB approval, to investigate whether triclosan-coated sutures lower the incidence of SSI and do not elicit more local or systemic adverse reactions than conventional sutures do. Early detection of surgical site infections or even periprosthetic joint infections is still one of the most challenging issues in the field of joint arthroplasty. Various measurements that were potential factors for detection should be investigated and documented. In this study, we used ESR, CRP, and IL-6 as well as surface temperature to test the efficacy of antibacterial sutures. Our data suggested that triclosan-coated sutures prevented an increase in IL-6 levels. In addition, the local skin temperature of the knees was lower in the triclosan group than in the control group at postoperative 3 months. Our study provided the information of IL-6 and surface temperature in the primary total knee arthroplasty, while the literature is sparse on this topic. Second, the rigorous follow-up of the patients might have raised patient awareness about their wound conditions. Most importantly, we believed that using PJI as the endpoint of analysis by avoiding the use of a theoretically better material was unethical. Fortunately, none of our patients developed a PJI. Whether a less strict follow-up in naïve patients would result in more wound complications is unknown. Third, the definition of superficial surgical site infection is limited to skin involvement only. Other surgical site infections are more serious and can involve tissues under the skin, organs, or implanted material. The measurements we used in the present study are indeed not convincing to discriminate these complications, especially, subcutaneous fatty tissue necrosis. Further fundamental studies to discriminate SSI, subcutaneous fatty necrosis, or even early PJI using tools such as thermal imaging system or even thermal-sensitive dressing should be considered.

## 5. Conclusion

Triclosan-coated sutures did not cause adverse local or systemic reactions: similar changes in serial inflammatory response occurred in both groups. Furthermore, falling levels of IL-6 imply that triclosan-coated sutures had a positive effect on postoperative knee inflammation. A more sensitive analytical measurement tool is needed to investigate local and systemic complications, especially in the early subclinical stage.

## Figures and Tables

**Table 1:** Patient demographic characteristics.

Characteristic	Overall	Triclosan group	Control group	*p* ^†^
	(*n* = 102)	(*n* = 51)	(*n* = 51)	
Gender (female)	76	36	40	
Age (years) (mean ± SD)	70.7 ± 7.4	71.3 ± 7.7	70.0 ± 7.1	0.372
Weight (kg) (mean ± SD)	67.6 ± 10.7	69.4 ± 11.5	65.9 ± 9.7	0.151
Height (cm) (mean ± SD)	154.1 ± 8.5	155.4 ± 7.7	153.0 ± 9.0	0.105
Surgery site: (left knee)	47	22	25	0.883^‡^

SD: standard deviation; ^†^independent *t*-test, unless otherwise stated; ^‡^Pearson *χ*^2^ test.

**Table 2:** Perioperative variables.

Variable	Triclosan group	Control group	*p* ^†^
	(*n* = 51)	(*n* = 51)	
Surgery duration (min) (mean ± SD)	125 ± 24	136 ± 34	0.046^*∗*^
Incision length (cm) (mean ± SD)	13.0 ± 1.3	12.8 ± 1.4	0.285
Blood loss (mL) (mean ± SD)	137.3 ± 37.2	150.0 ± 60.8	0.205
Perioperative complications	0	0	
Postoperative complications	0	2	0.495^‡^

SD: standard deviation; ^†^independent *t*-test, unless otherwise stated; ^‡^Pearson *χ*^2^ test; ^*∗*^*p* < 0.05.

**Table 3:** Summary of functional parameters in triclosan group and control group.

VAS / KSS / SF-12	Triclosan group	Control group	*p* ^†^
	(*n* = 51)	(*n* = 51)	
Baseline (preoperative value)			
VAS score	6.6 ± 1.7	7.0 ± 1.8	0.280
KSS score	40.8 ± 9.8	42.4 ± 10.6	0.424
SF-12 score	39.7 ± 18.1	40.9 ± 17.4	0.739
Postoperative day 1			
VAS score	8.6 ± 1.0	8.1 ± 0.9	0.017^*∗*^
Postoperative day 3			
VAS score	6.7 ± 1.0	6.4 ± 0.8	0.131
Postoperative 2 weeks			
VAS score	5.1 ± 1.6	4.6 ± 1.5	0.104
KSS score	45.6 ± 10.1	48.7 ± 9.7	0.126
SF-12 score	12.2 ± 29.4	10.2 ± 22.5	0.705
Postoperative 4 weeks			
VAS score	3.4 ± 1.5	3.4 ± 1.9	0.974
KSS score	55.5 ± 8.8	55.1 ± 9.3	0.834
SF-12 score	46.6 ± 33.9	49.5 ± 33.0	0.666
Postoperative 3 months			
VAS score	1.7 ± 1.5	1.5 ± 1.5	0.447
KSS score	61.7 ± 7.4	63.2 ± 6.2	0.279
SF-12 score	76.7 ± 30.9	80.3 ± 24.3	0.525

SD: standard deviation; VAS: visual analog scale; KSS: Knee Society Score; SF-12: Short Form 12; ^†^independent *t*-test, unless otherwise stated;  *χ*^2^ test; ^*∗*^*p* < 0.05.
